# Supplementary material for: Quantifying Tumor Heterogeneity via MRI Habitats to Characterize Microenvironmental Alterations in HER2+ Breast Cancer
Source: Cancers (Basel). 2022 Apr 6;14(7):1837. doi: 10.3390/cancers14071837 (PMC8997932; doi:10.3390/cancers14071837)
Supplement: Supplementary file 1 [file cancers-14-01837-s001.zip › cancers-1612116-supplementary.pdf]

Supplemental Materials

Supplemental Methods

Magnetic resonance imaging

Tumors were imaged using a 7 T preclinical MRI scanner (Bruker, Billerica, MA) equipped with a 40 mm transmit-receive volume coil (Bruker, Billerica, MA). Animal respiration was monitored, and body temperature maintained at 37º C by flowing warm air directly into the bore of the magnet. Each animal was placed in a custom-made animal bed with the tumor aligned to the isocenter of the magnet. For DW-MRI, image acquisition was respiratory triggered using a pneumatic pillow, placed between the mouse abdomen and animal bed, to reduce image artifacts due to bulk motion. A 5 mm diameter tube filled with water was placed along the length of the mouse and was used to verify the integrity of *ADC* measurements for each DW-MRI scan.

MRI processing

For each exam, the high-resolution T_2-weighted image was used to manually draw the tumor region-of-interest (ROI) around the tumor boundary for all slices containing tumor. ROIs were modified manually to exclude regions where no T_1-weighted or T_2-weighted signal was observed, suggestive of calcifications, as calcifications have been previously demonstrated to have low signal in MRI [1] (Figure S2).

The two acquisitions of DW-MRI data were averaged to generate the final signal intensity data used to calculate the *ADC* values. If significant motion artifacts were observed in a single acquisition, thereby limiting the accuracy of the estimated *ADC* values, that acquisition was removed from analysis. Parametric maps of the *ADC* were calculated by fitting signal intensity data from the DW-MRI data, acquired at three *b* values, to Eq. (S1), using a nonlinear least square optimization method (*lsqcurvefit*, MATLAB, Mathworks, Natick, MA):

$$S(b) = S_0 \exp(-ADC \cdot b), \qquad \text{(S1)}$$

where S_0 and $S(b)$ are the measured signal intensities before and after application of diffusion gradients, respectively, of strength b. If the signal intensities at the three b-values did not decrease monotonically (i.e.,

$S(b = 800) < S(b = 500) < S(b = 150)$), then the voxel was removed from subsequent analysis. The average (and 95% confidence interval) percent of tumor voxels removed was 6.9% (1.3%) per DW-MRI scan. Water phantoms were observed to have a mean (and 95% confidence interval) *ADC* of $2.5 \times 10^{-3} \pm 2.1 \times 10^{-5}$ mm^2/s.

The two acquisitions of T_1 mapping data (segFLASH) were averaged to generate the final signal intensity data used to calculate the native (i.e., pre-contrast) T_1 values. If significant motion artifacts were observed in a single repetition, that repetition was removed from analysis. The T_1 relaxation curve is described by Eq. (S2):

$$S(TI) = A - B\exp\left(-TI/T_1^*\right), \qquad \text{(S2)}$$

where $S(TI)$ is the signal intensity data at each inversion time, TI. Additionally, $A = (S_0\ T_1^*)/T_1$, and $B = S_0(1 + T_1^*/T_1)$, with S_0 the signal intensity at equilibrium, and T_1^* is described by

$$T_1^* = T_1/\left(\frac{B}{A} - 1\right). \qquad \text{(S3)}$$

T_1 maps were calculated using a three-parameter fit (A, B, and T_1) of Eq. (S2) to the segFLASH data using a nonlinear least square optimization method. Using native T_1 maps, signal intensity time courses from the DCE-MRI data were voxel-wise fit to the fast exchange regime (FXR) model [2,3], yielding estimates of K^{trans} (volume transfer constant), v_e (extravascular extracellular volume fraction), and τ_i (the average intracellular water lifetime). The FXR model incorporates a nonlinear relationship between the time-varying longitudinal relaxation time, $T_1(t)$, and the concentration of contrast agent within the tissue, $C_t(t)$, *via* Eq. (S4):

$$R_1(t) = \frac{1}{2}\left(2R_{1i} + r_1 C_t(t) + \frac{R_{10} - R_{1i} + 1/\tau_i}{v_e/f_w}\right) + \frac{1}{2}\left(\left(\frac{2}{\tau_i} - r_1 C_t(t) - \frac{R_{10} - R_{1i} + 1/\tau_i}{v_e/f_w}\right)^2 + 4\frac{\left(1 - v_e/f_w\right)}{\tau_i^2\left(v_e/f_w\right)}\right)^{1/2}, \qquad \text{(S4)}$$

where $R_1(t) = 1/T_1(t)$, R_{1i} is the intracellular T_1, R_{10} ($\equiv 1/T_{10}$) is the native R_1, r_1 is the longitudinal relaxivity of the contrast agent, and f_w is the fraction of water that is accessible to mobile contrast agent, which is set equal to 1 in this study. The $C_t(t)$ time course is modeled with the Kety-Tofts pharmacokinetic model [4,5]:

$$C_t(t)=K^{trans}\int_0^t C_p(u)\exp\left(-\left(\frac{K^{trans}}{v_e}\right)(t-u)\right)du, \qquad \text{(S5)}$$

using a population-derived arterial input function (AIF) [6], $C_p(t)$, to estimate K^{trans}, and v_e. Prior to fitting for pharmacokinetic parameters, the AIF was calibrated to segmented skeletal muscle tissue [7] for each DCE-MRI scan, to reduce systematic errors in the model fits. The AIF was calibrated by fitting an average signal intensity time course derived from muscle tissue to the Kety-Tofts model, and iteratively scaling the AIF until the v_e equaled a physiologically relevant value (i.e., 0.11) [7]. The bolus arrival-time was determined for each voxel, by fitting each voxel time course to a half-logistic function. The calibrated AIF was then shifted on a voxel-wise basis to align the enhancement time of $C_p(t)$ with the estimated bolus arrival-time [8]. Voxel-wise fits to the FXR model were bound within the physiological range for each parameter (i.e., 0 to 5 min^{-1} for K^{trans}, 0 to 1 for v_e, and 0 to 5 s for τ_i). The efflux constant, k_{ep}, was calculated as K^{trans}/v_e, with values beyond the physiological range (i.e., if $k_{ep} < 0\ \text{min}^{-1}$ or $k_{ep} > 5\ \text{min}^{-1}$) removed from analysis. The average (and 95% confidence interval) percent of tumor voxels removed was 2.2% (0.7%) per DCE scan. If contrast agent delivery was unsuccessful (due to catheter failure), then the DCE-MRI data was not used in the study (N=17). For parity with previous work [9], τ_i was not included in subsequent clustering analysis.

Immunofluorescence (IF) staining

For IF analysis, slides were blocked to prevent non-specific antibody binding using Biocare Blocking Reagent (Biocare Medical, LLC, Pacheco, CA), then stained with primary antibodies for 2 hours. Samples were stained with anti-CD31 (R&D Systems, Minneapolis, MN), anti-pimonidazole (Hypoxyprobe, Inc., Burlington, MA), anti-αSMA, anti-CD45, or anti-F4/80 (Abcam, Cambridge, UK). Samples were then stained with fluorophore-conjugated secondary antibodies for 40 minutes, before the addition of DAPI to stain cell nuclei, after which incubation was continued for 20 minutes. Primary and secondary antibody pairs are detailed in Table S1. Whole-slide images were then acquired at 10× resolution

using a Vectra Polaris Imaging System (Akoya Biosciences, Marlborough, MA). Representative IF images are shown in Figure S5.

Immunohistochemistry (IHC) and immunofluorescence (IF) image processing

Whole slide images from IHC and IF analyses were processed using Bio-Formats (http://openmicroscopy.org) and custom-built MATLAB scripts. A *k*-means clustering algorithm was used for automated segmentation of the H&E stained sections to generate masks for nuclei, necrosis, and non-tissue (gray) areas across the entire tumor slice [10]. Masks of the whole tissue area were semi-automatically generated using non-tissue area masks and manually drawn ROIs. Tissue necrosis was then quantified as a percentage of whole tissue area. Prior to IF image analysis, whole slide images were spectrally unmixed using inForm software (Akoya Biosciences, Marlborough, MA). A manually-selected threshold was applied to images to generate stain-positive masks for each stain: DAPI, CD31, αSMA, pimonidazole, CD45 and F4/80. Masks of viable tissue area were manually generated using DAPI stain-positive masks and manually drawn ROIs. Stain-positive regions were quantified as a percentage of viable tissue area. Pimonidazole was used to measure tissue hypoxia, and CD31 and αSMA were used to determine the vascular maturation index (VMI; [11]) was calculated using Eq. (S6):

$$\text{VMI} = \frac{\text{percent of viable tissue stained CD31+}}{\text{percent of viable tissue stained }\alpha\text{SMA+}}. \qquad \text{(S6)}$$

Macrophage infiltration was measured using CD45 and F4/80 co-staining (CD45, F4/80), with co-stain positive regions quantified as a percentage of viable tissue area.

Supplemental Table

Primary Antibody	Company	Secondary Antibody	Antigen Distribution*
anti-mouse CD31 (goat)	R&D Systems Cat. No. AF3628	Alexa Fluor 488 anti-goat IgG	Murine endothelial cells, platelets, granulocytes, macrophages, dendritic cells
anti-pimonidazole (mouse)	Hypoxyprobe Inc. Cat. No. HP1-XXX	Alexa Fluor 647 anti-mouse IgG	Binds to pimonidazole, which forms adducts with thiol containing proteins in cells that have an oxygen concentrations < 14 μM
anti-mouse αSMA (rabbit)	Abcam Cat. No. ab5694	Alexa Fluor 594 anti-rabbit IgG	Murine smooth muscle cells
anti-mouse CD45 (mouse)	Abcam Cat. No. ab3392	Alexa Fluor 647 anti-mouse IgG	Murine hematopoietic cells, except mature erythrocytes and platelets
anti-mouse F4/80 (rat)	Abcam Cat. No. ab6640	Alexa Fluor 594 anti-rat IgG	Murine macrophages, dendritic cells

* This study used athymic nude mice, which lack T-cells and B-cells. Hence, these cell types are excluded from this table.

Table S1: Primary and secondary antibodies for immunofluorescence staining. Listed is each primary antibody, its company and catalog number, the corresponding secondary antibody and the common cell types to which the primary antibody binds. Antigen distribution information was retrieved from product webpages from the company.

Supplemental Figures

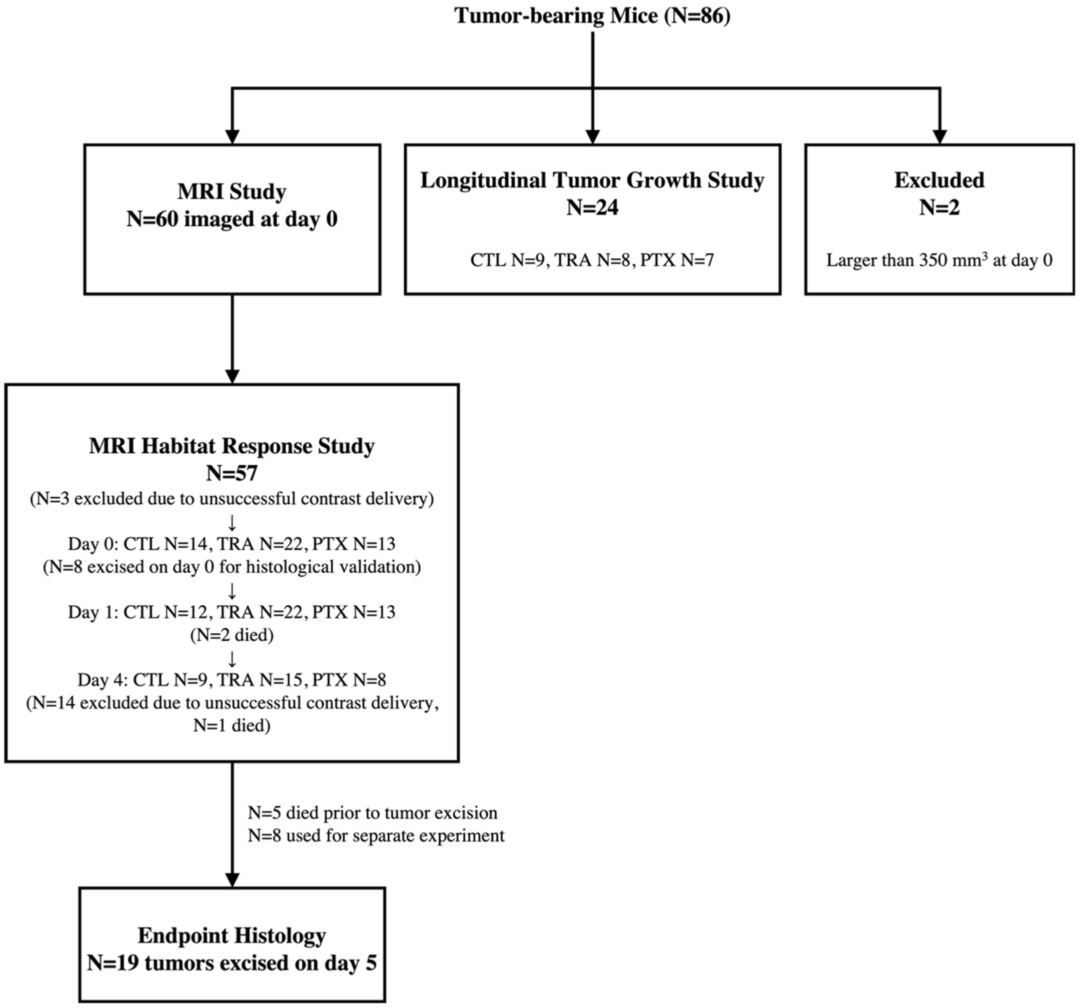

Figure S1. Study sample numbers across cohort treatment groups. The first row shows the number of mice used for the MRI and tumor growth studies, N=60 and N=24, respectively. The second row shows the number of mice per treatment group imaged at each time point (day 0, 1, 4) that were included in the habitat analysis. The final row shows the number of mice used for histological analysis at day 5. CTL: saline control, TRA: trastuzumab (10 mg/kg), PTX: paclitaxel (10 mg/kg).

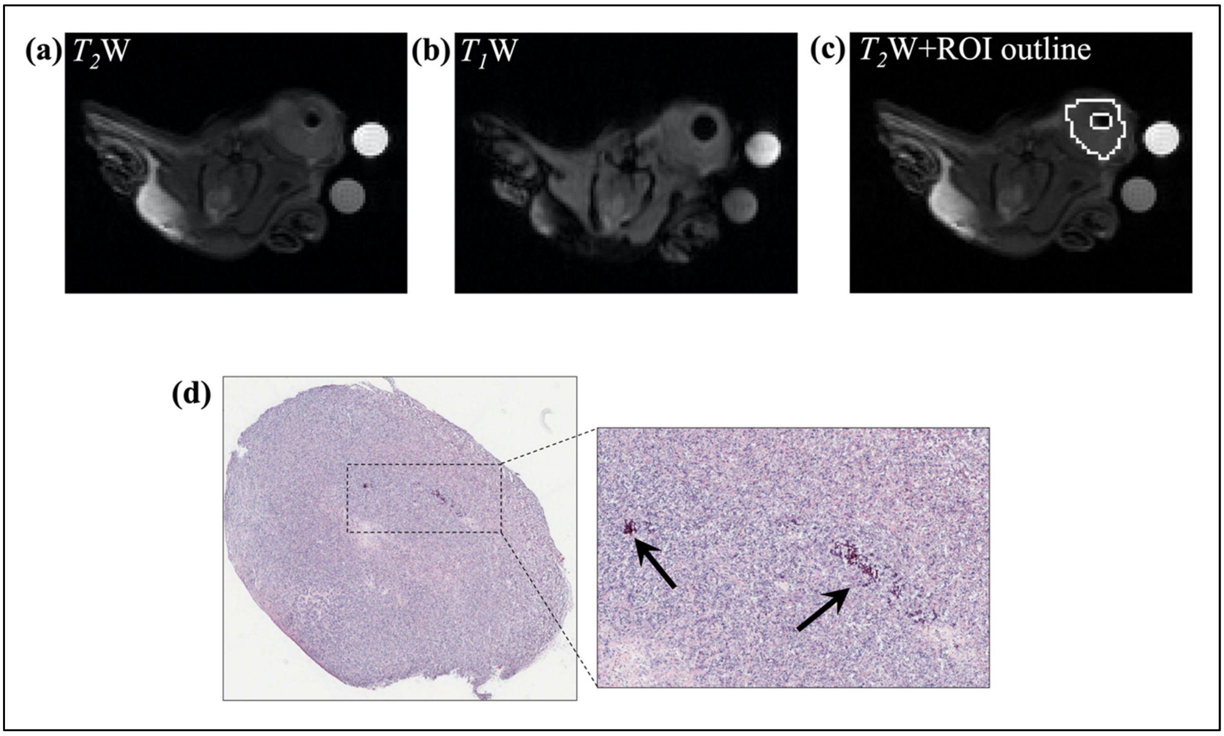

Figure S2. Example T_2-weighted (T_2W; panel a) and T_1-weighted (T_1W; panel b) images of a BT-474 tumor, with the voxels showing signal-voids removed from within the ROI. Panel c (T_2W + ROI outline) shows the outline of the final ROI, which excludes signal-void voxels from analysis. Panel d shows the corresponding H&E section for this tumor with arrows indicating calcifications, the potential source of the susceptibility artifact.

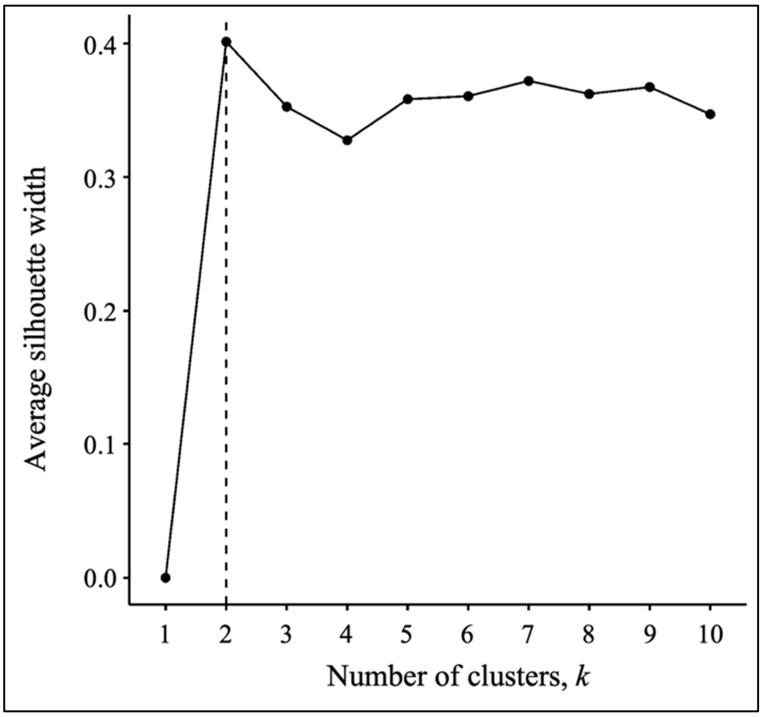

Figure S3. Silhouette analysis revealed the optimal number of tumor imaging phenotypes is two. The silhouette width is a measure of how close each observation (e.g., tumor) within a cluster is to objects within its own cluster, as well as other clusters. It can range from -1 to 1, with well-clustered observations having silhouette widths closer to 1. The average silhouette width can then be taken across all observations to determine the optimal number of clusters to be used in a clustering routine. The above plot indicates that the optimal number of clusters is two, yielding two tumor imaging phenotypes.

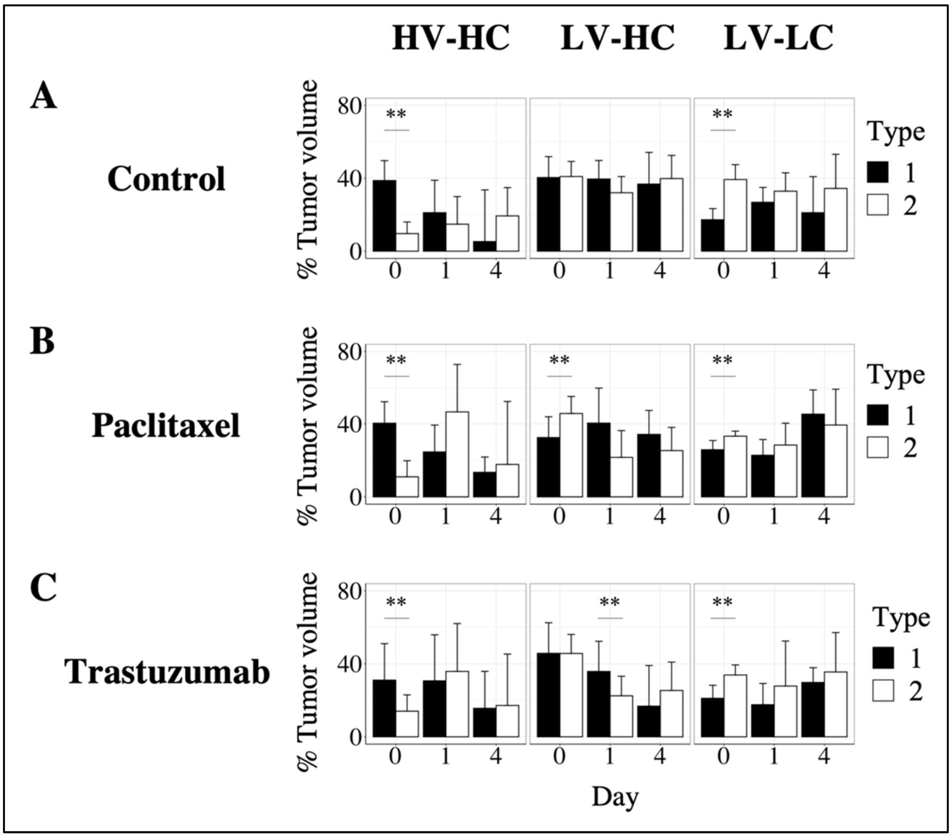

Figure S4. Differences in tumor composition between tumor imaging phenotypes. All treatment groups showed significant differences in percent tumor volume of HV-HC and LV-LC habitats between Type 1 and Type 2 tumors at day 0 (A-C, $p < 0.01$). Additionally, at day 0, Type 2 tumors within the paclitaxel treatment group also showed significantly higher percent tumor volume of the LV-HC habitat compared to Type 1 tumors ($p < 0.01$). Type 2 tumors treated with trastuzumab showed decreased percent tumor volume of the LV-HC habitat compared to trastuzumab treated Type 1 tumors at day 1 ($p < 0.05$). No other significant differences between tumor phenotypes in percent tumor volume of habitats was observed for control, paclitaxel, or trastuzumab-treated tumors ($p > 0.05$).

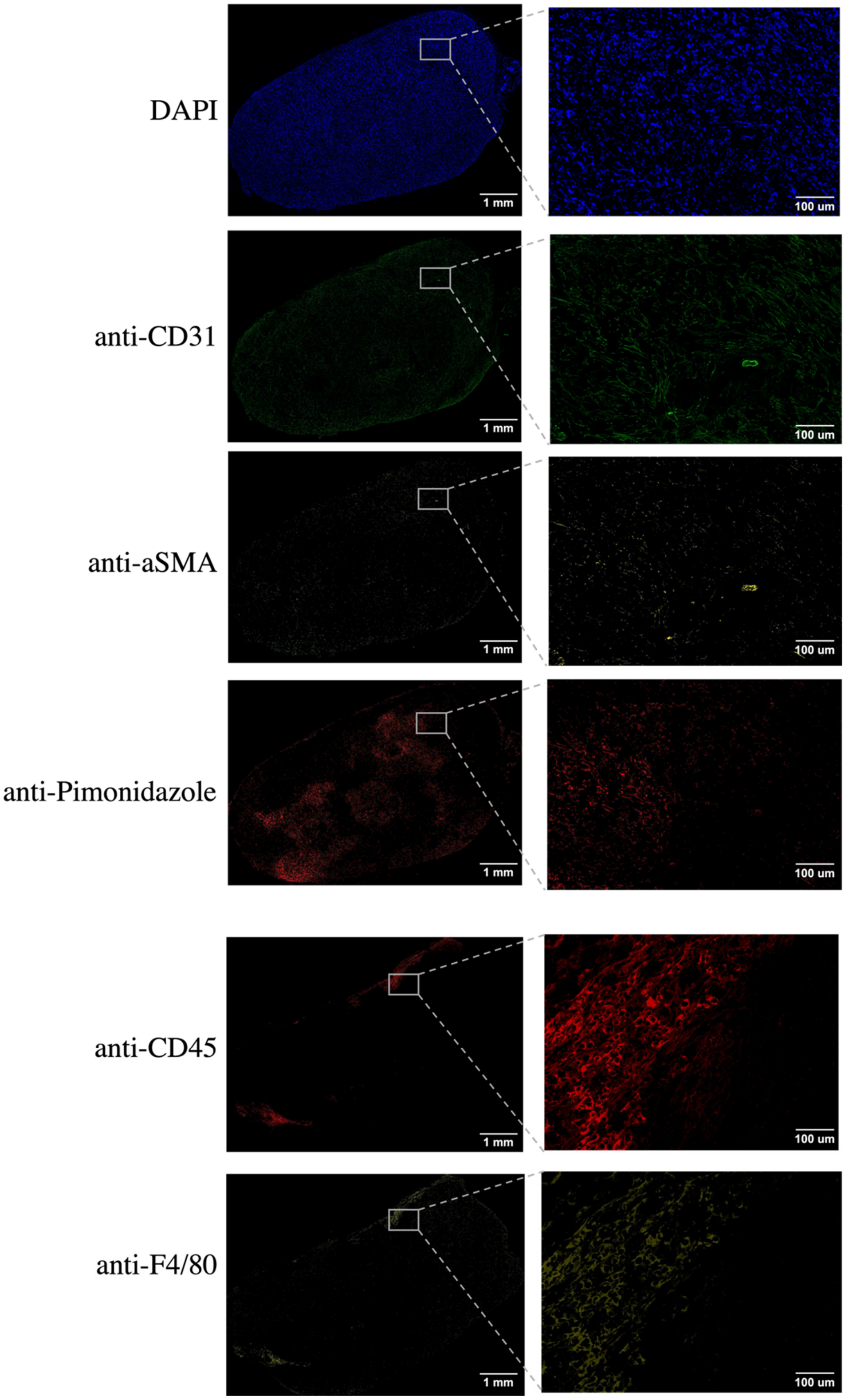

Figure S5. Representative immunofluorescence images. From top to bottom, shown are representative DAPI, anti-CD31, anti-aSMA, anti-pimonidazole, anti-CD45, and anti-F4/80 stains. On the left are whole slice images of a tumor cross section, and the right shows a zoomed in region within the slice.

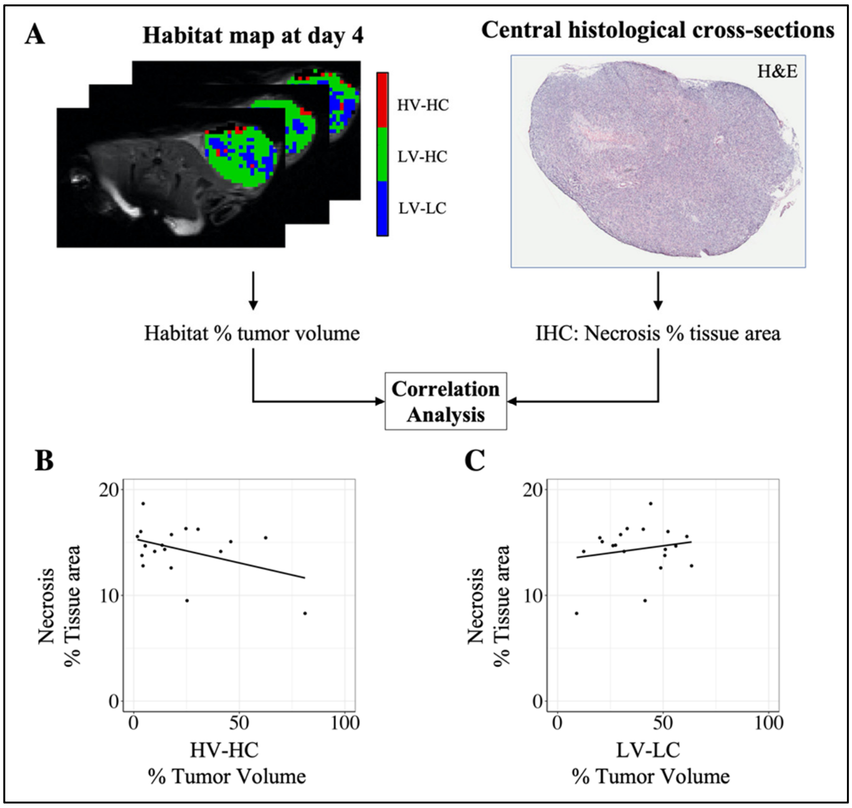

Figure S6. Correlation between MRI tumor habitats and necrosis percent tissue area. For each tumor, the percent tumor volume of each habitat was calculated (panel A). For each immunohistochemistry (IHC) tumor section regions of necrosis were segmented and quantified as percent tissue area (panel A). The correlation was calculated between percent tumor volume of each habitat and percent tissue area of necrosis. Percent tumor volume of the HV-HC habitat showed a negative correlation (trending towards significance) with necrosis percent tissue area (r^2 = -0.47, p = 0.05, panel B). Percent tumor volume of the LV-LC habitat showed no significant correlation with necrosis percent tissue area (r^2 = 0.24, p = 0.33, panel C).

References

1. Wehrli, F.W. Magnetic Resonance of Calcified Tissues. *J. Magn. Reson.* **2013**, *229*, 35–48, doi:10.1016/j.jmr.2012.12.011.
2. Yankeelov, T.E.; Rooney, W.D.; Li, X.; Springer, C.S. Variation of the Relaxographic "Shutter-Speed" for Transcytolemmal Water Exchange Affects the CR Bolus-Tracking Curve Shape. *Magn. Reson. Med.* **2003**, *50*, 1151–1169, doi:10.1002/mrm.10624.
3. Yankeelov, T.E.; Rooney, W.D.; Huang, W.; Dyke, J.P.; Li, X.; Tudorica, A.; Lee, J.-H.; Koutcher, J.A.; Springer, C.S. Evidence for Shutter-Speed Variation in CR Bolus-Tracking Studies of Human Pathology. *NMR Biomed.* **2005**, *18*, 173–185, doi:10.1002/nbm.938.
4. Kety, S.S. The Theory and Applications of the Exchange of Inert Gas at the Lungs and Tissues. *Pharmacol. Rev.* **1951**, *3*, 1–41.
5. Tofts, P.S. Modeling Tracer Kinetics in Dynamic Gd-DTPA MR Imaging. *J. Magn. Reson. Imaging* **1997**, *7*, 91–101, doi:10.1002/jmri.1880070113.
6. Loveless, M.E.; Halliday, J.; Liess, C.; Xu, L.; Dortch, R.D.; Whisenant, J.; Waterton, J.C.; Gore, J.C.; Yankeelov, T.E. A Quantitative Comparison of the Influence of Individual versus Population-Derived Vascular Input Functions on Dynamic Contrast Enhanced-MRI in Small Animals. *Magn. Reson. Med.* **2012**, *67*, 226–236, doi:10.1002/mrm.22988.
7. Li, X.; Rooney, W.D.; Várallyay, C.G.; Gahramanov, S.; Muldoon, L.L.; Goodman, J.A.; Tagge, I.J.; Selzer, A.H.; Pike, M.M.; Neuwelt, E.A.; et al. Dynamic Contrast Enhanced-MRI with Extravasating Contrast Reagent: Rat Cerebral Glioma Blood Volume Determination. *J. Magn. Reson. San Diego Calif 1997* **2010**, *206*, 190–199, doi:10.1016/j.jmr.2010.07.004.
8. Wu, C.; Pineda, F.; Hormuth, D.A.; Karczmar, G.S.; Yankeelov, T.E. Quantitative Analysis of Vascular Properties Derived from Ultrafast DCE-MRI to Discriminate Malignant and Benign Breast Tumors. *Magn. Reson. Med.* **2019**, *81*, 2147–2160, doi:10.1002/mrm.27529.
9. Syed, A.K.; Whisenant, J.G.; Barnes, S.L.; Sorace, A.G.; Yankeelov, T.E. Multiparametric Analysis of Longitudinal Quantitative MRI Data to Identify Distinct Tumor Habitats in Preclinical Models of Breast Cancer. *Cancers* **2020**, *12*, 1682, doi:10.3390/cancers12061682.
10. Syed, A.K.; Woodall, R.; Whisenant, J.G.; Yankeelov, T.E.; Sorace, A.G. Characterizing Trastuzumab-Induced Alterations in Intratumoral Heterogeneity with Quantitative Imaging and Immunohistochemistry in HER2+ Breast Cancer. *Neoplasia* **2019**, *21*, 17–29, doi:10.1016/j.neo.2018.10.008.
11. Vangestel, C.; Wiele, C.V. de; Damme, N.V.; Staelens, S.; Pauwels, P.; Reutelingsperger, C.P.M.; Peeters, M. 99mTc-(CO)3 His-Annexin A5 Micro-SPECT Demonstrates Increased Cell Death by Irinotecan During the Vascular Normalization Window Caused by Bevacizumab. *J. Nucl. Med.* **2011**, *52*, 1786–1794, doi:10.2967/jnumed.111.092650.
